# Identification of Domains and Amino Acids Essential to the Collagen Galactosyltransferase Activity of GLT25D1

**DOI:** 10.1371/journal.pone.0029390

**Published:** 2011-12-21

**Authors:** Claire Perrin-Tricaud, Christoph Rutschmann, Thierry Hennet

**Affiliations:** Institute of Physiology, University of Zürich, Zürich, Switzerland; Consejo Superior de Investigaciones Cientificas, Spain

## Abstract

Collagen is modified by hydroxylation and glycosylation of hydroxylysine residues. This glycosylation is initiated by the β1,O galactosyltransferases GLT25D1 and GLT25D2. The structurally similar protein cerebral endothelial cell adhesion molecule CEECAM1 was previously reported to be inactive when assayed for collagen glycosyltransferase activity. To address the cause of the absent galactosyltransferase activity, we have generated several chimeric constructs between the active human GLT25D1 and inactive human CEECAM1 proteins. The assay of these chimeric constructs pointed to a short central region and a large C-terminal region of CEECAM1 leading to the loss of collagen galactosyltransferase activity. Examination of the three DXD motifs of the active GLT25D1 by site-directed mutagenesis confirmed the importance of the first (amino acids 166–168) and second motif (amino acids 461–463) for enzymatic activity, whereas the third one was dispensable. Since the second DXD motif is incomplete in CEECAM1, we have restored the motif by introducing the substitution S461D. This change did not restore the activity of the C-terminal region, thereby showing that additional amino acids were required in this C-terminal region to confer enzymatic activity. Finally, we have introduced the substitution Q471R-V472M-N473Q-P474V in the CEECAM1-C-terminal construct, which is found in most animal GLT25D1 and GLT25D2 isoforms but not in CEECAM1. This substitution was shown to partially restore collagen galactosyltransferase activity, underlining its importance for catalytic activity in the C-terminal domain. Because multiple mutations in different regions of CEECAM1 contribute to the lack of galactosyltransferase activity, we deduced that CEECAM1 is functionally different from the related GLT25D1 protein.

## Introduction

Collagen undergoes several post-translational modifications before formation of a right-handed triple helix in the endoplasmic reticulum. These modifications include the hydroxylation of selected proline [1] and lysine [2] residues. Thereafter, a portion of hydroxylysine (Hyl) residues are further modified by the addition of carbohydrates, forming the disaccharide Glc(α1-2)Gal(β1-O)Hyl [3], [4]. The extent of hydroxylation and glycosylation depends on the type of collagen and their tissue distribution. The formation of hydroxyproline is essential for the thermal stability of the collagen triple helix [5]. Lysyl hydroxylation is important for the cross-linking of collagen fibrils and acts as substrate for glycosylation reactions. The importance of collagen post-translational modifications is reflected by the diseases associated to defective collagen modifications. Mutations in prolyl 3-hydroxylase and in the lysyl hydroxylase genes lead to various forms of skeletal dysplasia [6], [7],[8],[9].

Whereas the roles of proline and lysine hydroxyllation in collagen biology are well established, the functional relevance of collagen glycosylation is presently unclear. Recently, we have identified the two genes *GLT25D1* and *GLT25D2* as encoding collagen galactosyltransferase (ColGalT) enzymes [10]. RNA interference studies in *Caenorhabditis elegans* have shown that the inactivation of the lysyl hydroxylase gene *let-268* yields a lethal phenotype related to abnormal collagen type-IV secretion [11], [12]. Similarly, the inactivation of the *D2045.9* gene, the putative ortholog to human *GLT25D1* and *GLT25D2*, is associated to growth defects and multiple morphologic abnormalities [13], [14].

The sequences of the GLT25D1 and GLT25D2 proteins were found to be strongly similar to the cerebral endothelial cell adhesion molecuzle (CEECAM1) protein [15]. However, in spite of the strong structural conservation, CEECAM1 was found to be inactive when assayed for collagen glycosyltransferase activity [10]. Although several types of collagen acceptors have been tested, it could not be excluded that CEECAM1 only recognizes specific substrates. Alternatively, the claimed localization of CEECAM1 at the surface of endothelial cells [15] suggests that the protein does not function as a glycosyltransferase like GLT25D1 and GLT25D2 proteins. The CEECAM1 protein could be enzymatically inactive as collagen glycosyltransferase because of point mutations introduced during evolution. The polymorphism of ABO blood group glycosyltransferases [16] or the inactive form of the α1,3 galactosyltransferase gene in primate genomes [17] are typical examples of such lost glycosyltransferase activities.

In the present work, we investigate the rationale for the lack of ColGalT activity in the CEECAM1 protein by domain swapping and single point mutation. By permutation with portions of the active GLT25D1 galactosyltransferase, the production of GLT25D1-CEECAM1 chimeric proteins enables the identification of the regions responsible for the loss of galactosyltransferase activity. In parallel, this approach allows narrowing down the regions of GLT25D1 that are essential for the catalytic activity.

## Results

**Figure 1:**
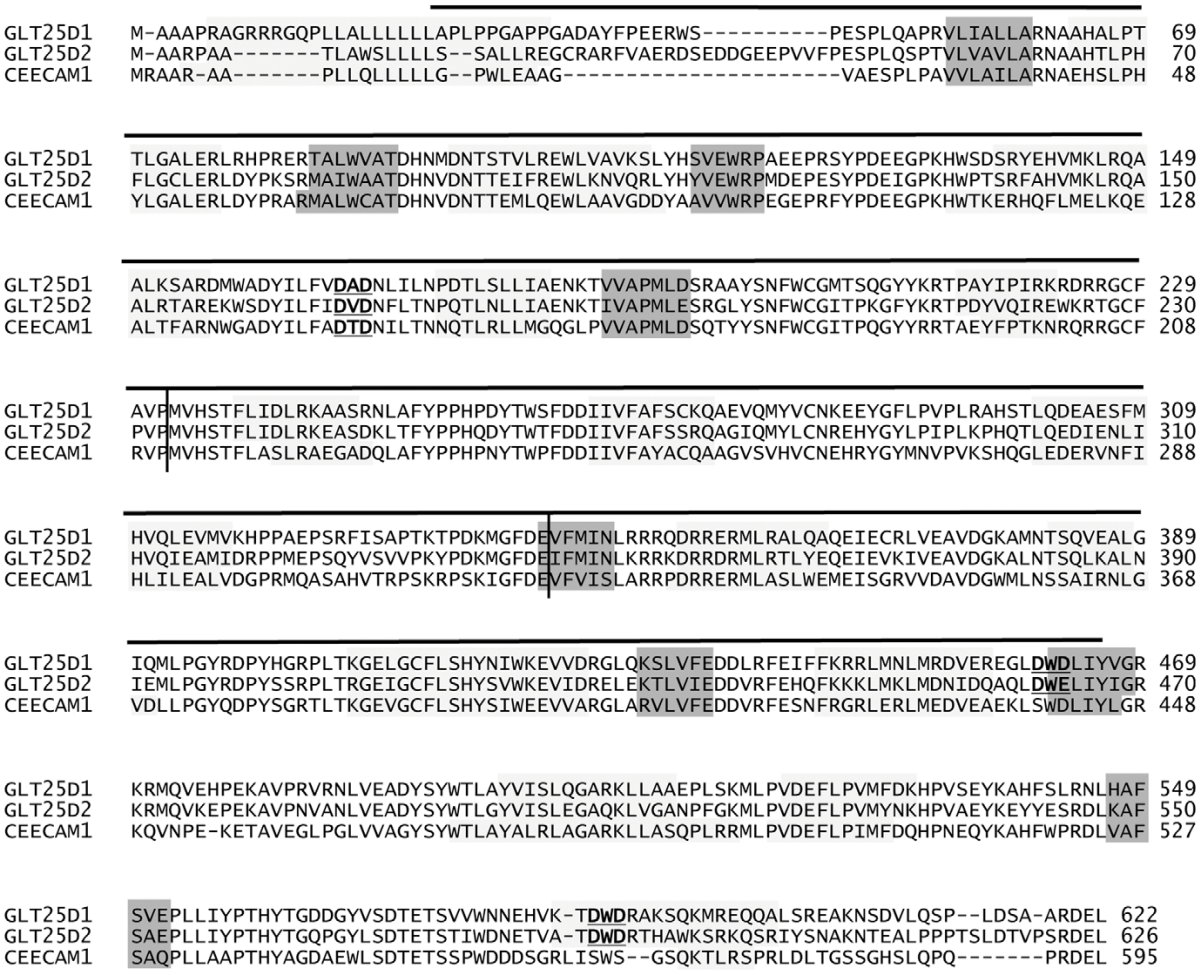
Structural prediction of GLT25D1, GLT25D2 and CEECAM1 proteins. The protein sequences of human GLT25D1 (Swiss-Prot:Q8NBJ5), GLT25D2 (Swiss-Prot:Q81IYK4) and CEECAM1 (Swiss-Prot:Q5T4B2) were aligned using the PROMALS3D program. The sequences were analyzed and separated in two closely related groups, the first one with GLT25D1-D2 and the second one with CEECAM1, as determined with an identity threshold of 0.6. The secondary structure predictions are marked by shaded blocks, with light grey representing α-helices and dark grey β-strands. The predicted N-terminal, central and C-terminal domains are demarcated by vertical lines. The DXD motifs are underlined and marked in bold. The region ranging from amino acids 24 to 466 of human GLT25D1 structurally related to the *Escherichia coli* chondroitin polymerase (PDB ID: 2z86) is marked with a horizontal line above the sequences.

The CEECAM1 protein is structurally similar to the ColGalT enzymes GLT25D1 and GLT25D2. The comparison of the three polypeptides shows 55% sequence identity between human CEECAM1 and human GLT25D1 and 50% between human CEECAM1 and human GLT25D2 [10]. The three proteins also share the same putative conformation and domain organization as predicted by the PROMALS3D algorithm [18] (Fig. 1). To further compare the three proteins, we aligned GLT25D1, GLT25D2 and CEECAM1 with known protein structures using the pdb70 database and HHpred tool (http://toolkit.tuebingen.mpg.de/hhpred) [19]. This search pointed to a potential structural similarity of the three proteins with the chondroitin polymerase from *Escherichia coli* (PDB ID: 2z86). The region ranging from amino acids 24 to 466 of human GLT25D1 produced a hit with an E-value of 6.2E-09 and P-value of 2.7E-13. This bacterial chondroitin polymerase belongs to the CAZY GT2 family of glycosyltransferases and contains two functional domains adopting a glycosyltransferase GT-A fold [20]. Such a binary structure is supposedly adopted by the GLT25D1, GLT25D2 and CEECAM1 proteins as also noted by Liefhebber et al. [21]. Based on this prediction, the GLT25D1, GLT25D2 and CEECAM1 proteins can be parted into three structural domains: a N-terminal domain of about 230 amino acids, a central domain of about 110 amino acids and a C-terminal domain of about 280 amino acids (Fig. 1).

**Figure 2:**
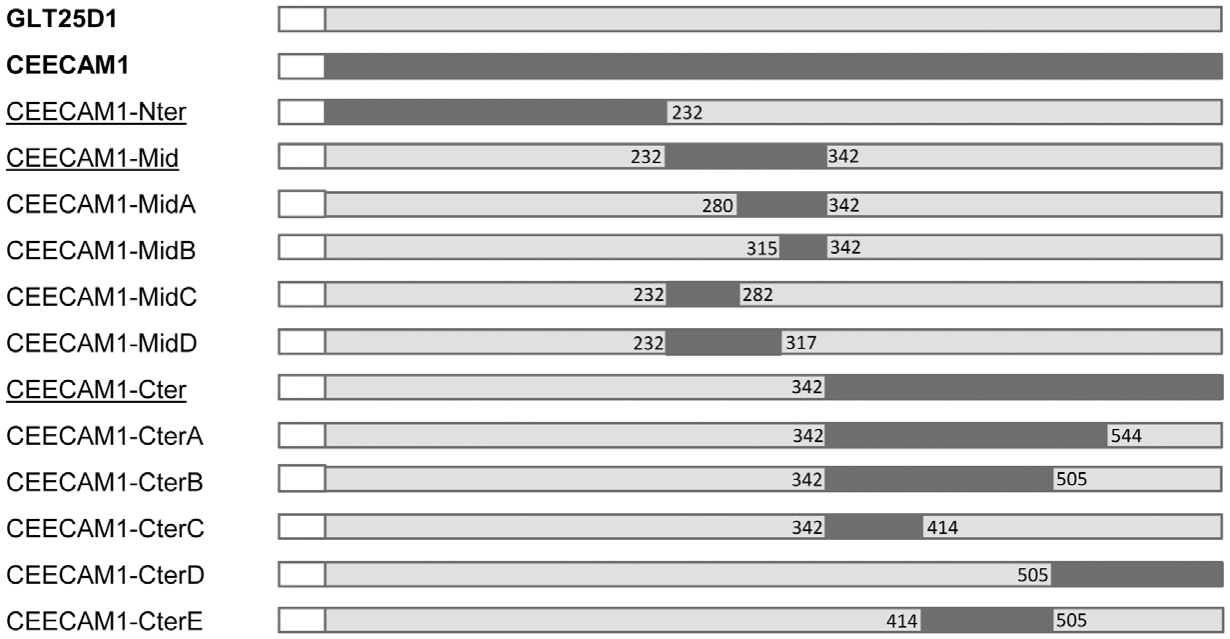
Schematic representation of chimeric GLT25D1-CEECAM1 constructs. Segments of GLT25D1 and CEECAM1 are marked in light grey and dark grey, respectively. The boundaries of each segment are given by the amino acid positions in the GLT25D1 protein. The Flag tag is shown with a white box at the N-terminus of each construct.

**Figure 3:**
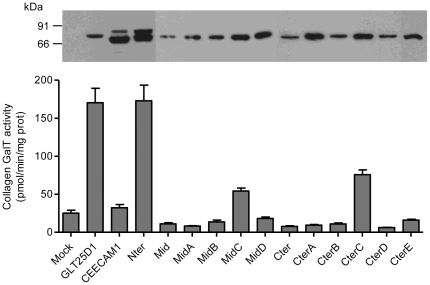
ColGalT activity of GLT25D1, CEECAM1 and chimeric constructs. Values represent the mean and S.E.M. of at least four independent assays per construct. The mock sample indicates the activity measured in Sf9 cells infected with an empty baculovirus. The protein expression of each construct is shown at the top of each bar as performed by Western blotting using an anti-Flag tag antibody.

To identify the molecular basis for the lack of collagen glycosyltransferase activity in CEECAM1, we constructed chimeric proteins between GLT25D1 and CEECAM1 based on the three domains mentioned here above. The corresponding chimeras were named CEECAM1-Nter, CEECAM1-Mid and CEECAM1-Cter (Fig. 2). The chimeric proteins were Flag-tagged at their N-terminus and expressed in Sf9 cells as recombinant baculoviruses. The amount of recombinant proteins present in Sf9 cell lysates was monitored by Western blotting using an anti-Flag antibody and ColGalT activity was assayed towards denatured bovine collagen type I. The CEECAM1 and CEECAM1-Nter proteins were expressed as two glycoforms, whereas the other chimeric proteins expressed as a single band (Fig. 3). The CEECAM1-Nter construct yielded the same ColGalT activity as the GLT25D1 enzyme, showing that the N-terminal domain of CEECAM1 does not contribute to the absence of glycosyltransferase activity. By contrast, the constructs including either the central or the C-terminal domain of CEECAM1 did not show any ColGalT activity (Fig. 3).

**Figure 4:**
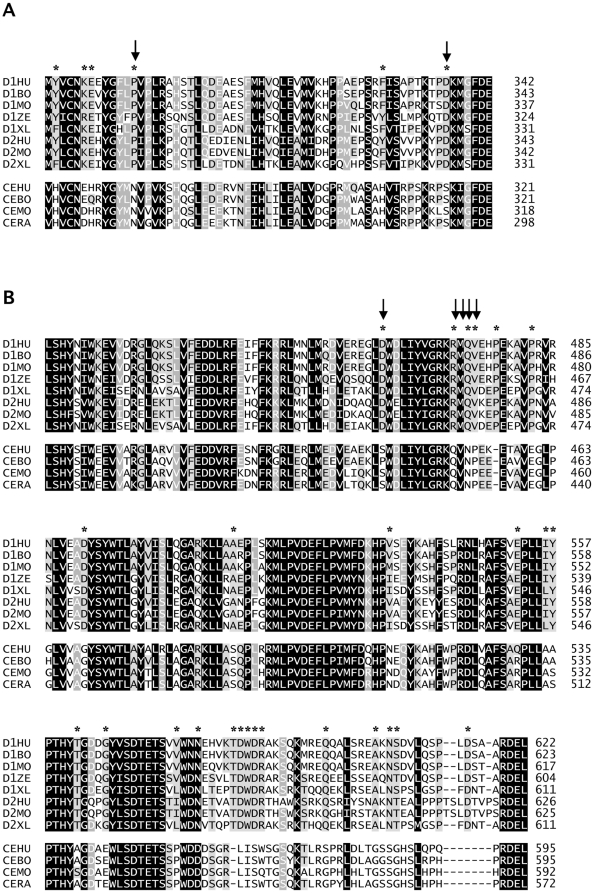
Multiple alignments of GLT25D1, GLT25D2 and CEECAM1. A) The portion of the central domain essential for ColGalT activity is represented encompassing GLT25D1 amino acids 280 to 342. Sequences of GLT25D1 human (D1HU; Swiss-Prot:Q8NBJ5), bovine (D1BO; Swiss-Prot:A5PK45), mouse (D1MO; Swiss-Prot:Q8K297), zebrafish (D1ZE; Swiss-Prot:A5PMF6), xenopus (D1XL; Swiss-Prot:A0JPH3) ; GLT25D2 human (D2HU; Swiss-Prot:Q81IYK4), mouse (D2MO; Swiss-Prot:Q6NVG7), xenopus (D2XL; Swiss-Prot:Q5U483); CEECAM1 human (CEHU; Swiss-Prot:Q5T4B2), bovine (CEBO; Swiss-Prot:A7MB73), mouse (CEMO; Swiss-Prot:A3KGW5), rat (CERA; Swiss-Prot:Q5U309) were aligned using ClustalW. Black squares represent amino acids identical or strongly similar in all proteins, dark grey squares represent amino acids identical or similar in at least 10 proteins, light grey squares represent amino acids identical or similar in at least 7 proteins. The amino acids conserved in GLT25D1 and GLT25D2 but not in CEECAM1 are marked with a star at the top of the alignment. The residues P292 and D336 are marked with arrows. B) The alignment shows the portion of the C-terminal domain essential for ColGalT activity corresponding to GLT25D1 amino acids 414 to 622. The origin of the sequences and the markings are the same as in A.

To delineate more precisely the regions of CEECAM1 and GLT25D1 that are essential for enzymatic activity, we have produced four additional chimeric constructs for the central domain and five additional constructs for the C-terminal domain. From the four chimeric constructs containing portions of the CEECAM1 central domain, only the construct, in which the stretch between amino acids 232 and 282 of GLT25D1 was replaced by the corresponding stretch of CEECAM1 (named CEECAM1-MidC), was found to be enzymatically active, yet with decreased ColGalT activity to 32% of wildtype GLT25D1 levels (Fig. 3). The analysis of these four chimeric constructs delimitated a CEECAM1 stretch of 63 amino acids that caused the loss of the enzymatic activity when introduced into GLT25D1. The comparison of GLT25D1, GLT25D2 and CEECAM1 protein sequences from different animal genomes showed that this stretch corresponds to a strongly conserved sequence region (Fig. 4A). Within this stretch of 63 amino acids, only six conserved residues within GLT25D1 and GLT25D2 isoforms were found to be different in CEECAM1.

To study the impact of the C-terminal domain on ColGalT activity, five chimeric constructs encompassing parts of the CEECAM1 C-terminal domain in GLT25D1 (Fig. 2) were expressed in Sf9 cells and assayed for enzymatic activity. This analysis pointed to a large portion of the C-terminal domain as being involved in the catalysis of the ColGalT reaction. In fact, only the GLT25D1 stretch between amino acids 342 and 414 was replaceable for enzymatic activity, as shown by the partially active construct CEECAM1-CterC (Fig. 3). The examination of the C-terminal domain of GLT25D1, GLT25D2 and CEECAM1 from multiple animal species revealed 26 amino acid residues strongly conserved in GLT25D1 and GLT25D2 but not in CEECAM1. Of interest, two DXD motifs are included among these diverging amino acids (Fig. 4B).

**Figure 5:**
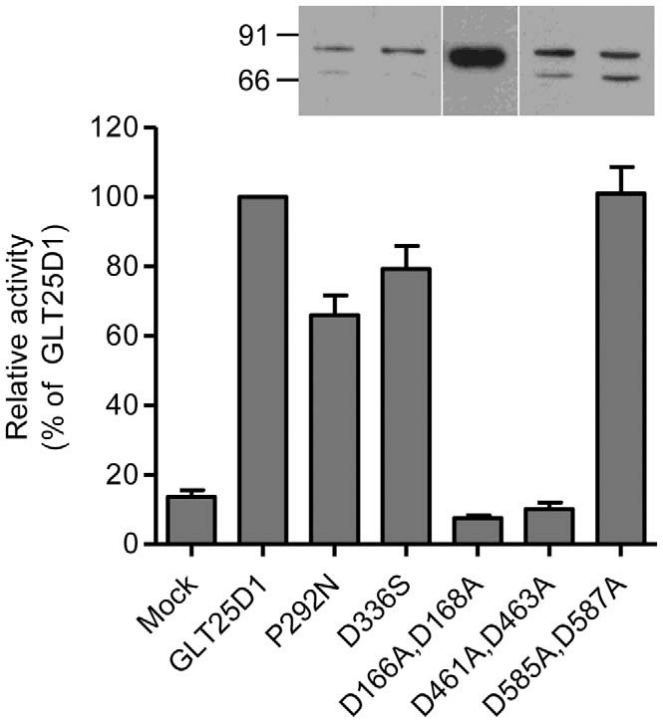
ColGalT activity of GLT25D1 mutant constructs. The enzymatic activity is expressed relatively to the activity of wildtype GLT25D1. Values show the means and S.E.M. of at least four independent assays. The expression of the mutant constructs is confirmed by Western blotting performed in Sf9 cell lysate using an anti-Flag antibody. The Mock sample shows the activity measured in Sf9 cells infected with an empty baculovirus.

The presence of DXD motifs is typical for glycosyltransferases utilizing nucleotide-activated sugars as donor substrates. Such DXD motifs are usually involved in the binding of the nucleotide diphosphate moiety through Mn^2+^ chelation [22]. The collagen galactosyltransferases GLT25D1 and GLT25D2 include three DXD motifs and one related EDD motif (Fig. 1). The EDD and one DXD motifs are also conserved in CEECAM1, whereas two C-terminally localized DXD motifs are missing in CEECAM1. This suggests that the C-terminal DXD motifs may be important for the activity of the GLT25D1, considering their absence in the enzymatically inactive CEECAM1 protein. To determine the importance of the DXD motifs on the activity of GLT25D1, we have produced mutant forms of GLT25D1 lacking each of these three motifs. The mutant GLT25D1 proteins mainly yielded two glycoforms (Fig. 5), although this pattern was not seen in all expressed samples. The substitution D166A-D168A led to an inactive protein, thus establishing the importance of the first DXD motif for the ColGalT activity. Note worthily, this DXD motif is also found in the N-terminal domain of CEECAM1. The second DXD motif of GLT25D1, found at D461-D463, was also essential to the ColGalT activity, since its mutation yielded an inactive GLT25D1 protein (Fig. 5). By contrast, the last DXD motif of GLT25D1 at D585-D587 was dispensable for the ColGalT activity (Fig. 5).

As noted in our domain swapping experiments, a portion of the CEECAM1 central domain abolished the enzymatic activity when replacing the GLT25D1 stretch encompassing amino acids 280 to 342 (Fig. 3). In an attempt to isolate amino acids in this central domain that are important for this enzyme activity, we replaced the residues P292 and D336 in GLT25D1 by the corresponding residues found in CEECAM1, thereby yielding the substitutions P292N and D336S (Fig. 4). These two residues were selected because they are strictly conserved in animal GLT25D1 and GL25D2 isoforms. In addition, P292 may be involved in making a critical twist in the polypeptide structure. The expression of the two P292N and D336S GLT25D1 mutants showed that these two substitutions only lightly decreased ColGalT activity, thereby speaking against a critical involvement of these amino acids in the inactivity of CEECAM1 (Fig. 5).

**Figure 6:**
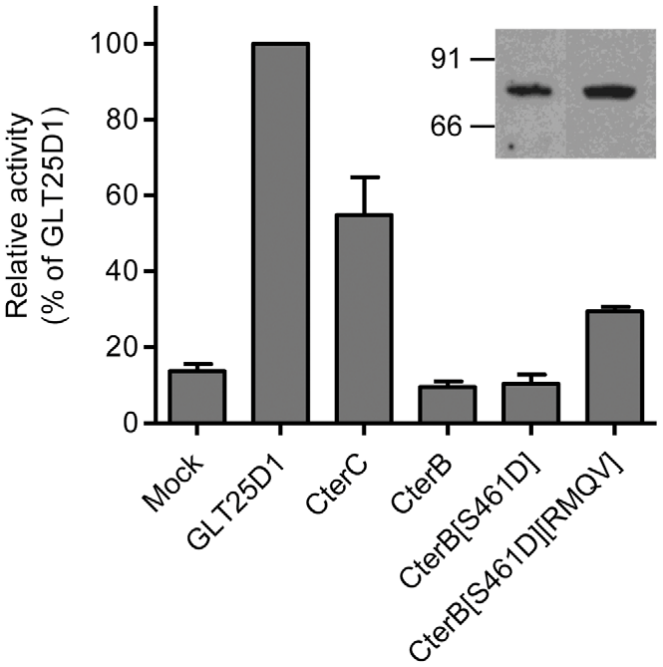
ColGalT activity of CEECAM1-CterB mutant constructs. The enzymatic activity is expressed relatively to the activity of wildtype GLT25D1. Values show the means and S.E.M. of at least four independent assays. The expression of the mutant constructs CEECAM1-CterB[S461D] and CEECAM1-CterB[S461D][RMQV] is confirmed by Western blotting performed in Sf9 cell lysate using an anti-Flag antibody. RMQV stands for the substitution Q471R-V472M-N473Q-P474V.

The comparison between the C-terminal domain of GLT25D1, GLT25D2 and CEECAM1 isoforms from various animal genomes pointed to 26 amino acids diverging between GLT25D1 and GLT25D2 on the one hand and CEECAM1 on the other hand (Fig. 4). As shown here above, the C-terminal domain includes an essential DXD motif that is not found in CEECAM1. We chose to introduce this DXD motif in CEECAM1 to determine if this change could restore the ColGalT activity in the inactive chimeric construct CEECAM1-CterB (Fig. 2). To this end, the S461D substitution was engineered and the resulting CEECAM1-CterB[S461D] construct assayed for enzymatic activity. This test showed that the S461D substitution was insufficient to restore ColGalT activity (Fig. 6). Another strictly conserved stretch found in GLT25D1 and GLT25D2 but absent in CEECAM1 is located just downstream of the D461–D463 DXD motif. This stretch begins at R471 and reads RMQV whereas it corresponds to a QVNP sequence in CEECAM1 (Fig. 4). We have added the substitution Q471R-V472M-N473Q-P474V in the CEECAM1-CterB[S461D] construct, yielding CEECAM1-CterB[S461D][RMQV]. This new mutant form showed a partially restored ColGalT activity (Fig. 6), thus demonstrating the importance of this stretch for the enzymatic activity.

Finally, we determined the apparent K_m_ and V_max_ parameters of the partially active chimeric constructs CEECAM1-MidC and CEECAM1-CterC to assess whether the decreased activity can be related to altered interaction with the substrates. This kinetic analysis showed that the low ColGalT activity of CEECAM1-MidC was due to decreased V_max_ (Table 1), suggesting that the central domain is important to maintain a normal reaction rate. By contrast, the analysis of the CEECAM1-CterC showed normal V_max_ values but increased K_m_ values for both donor and acceptor substrates (Table 1). This finding supports the idea that the C-terminal domain is important for the binding to UDP-Gal and to collagen.

**Table 1:** Kinetic parameters for chimeric constructs CEECAM1-MidC and CEECAM1-CterC.

	UDP-Gal		Collagen	
Enzyme	Km (µM)	Vmax (pmol min^−1^)	Km (µg µl^−1^)	Vmax (CPM min^−1^)
**GLT25D1**	29.91±2.56	12.45±1.09	5.7±0.9	235.13±25.66
**CEECAM1-MidC**	20.31±1.63	6.86±0.45	5.04±1.12	108.53±29.95
**CEECAM1-CterC**	74.29±5.56	11.2±1.4	13.4±1.75	259.27±63.48

Values represent the average ± S.E.M. of three independent experiments.

## Discussion

The present study demonstrates that the inactivity of CEECAM1 as a ColGalT is related to sequence variations in two distinct regions of the protein. Based on the analysis of chimeric constructs between the inactive CEECAM1 and the active GLT25D1 proteins, the study also enabled the identification of structural motifs essential to the ColGalT activity. The recombinant GLT25D1, CEECAM1 and chimeric proteins tested were expressed to similar levels in Sf9 insect cells and did not yield insoluble aggregates. The resulting uniform expression patterns enabled the functional comparison of all constructs described in the study.

The N-terminal domain of GLT25D1 and CEECAM1 were interchangeable without losing the galactosyltransferase activity, which did not mean that this domain was dispensable for the enzymatic activity. In fact, the DXD motif in this domain was shown to be essential to the activity of GLT25D1. Since the N-terminal domain of CEECAM1 could yield an active chimeric protein when combined with GLT25D1, the experiment showed that both proteins are structurally closely related and that such chimeric constructs are neither instable nor inactive *per se*. The N-terminal domain of GLT25D1, GLT25D2 and CEECAM1 also shared structural similarity with glycosyltransferases like the polypeptide N-acetylgalactosaminyltransferases-2, -10 (CAZY family GT27) and the α1,4 N-acetylhexosaminyltransferase EXTL2 (CAZY family GT64) [23], [24], [25], indicating that the sequence context around the first DXD motif is suitable for an interaction with Mn^2+^ and with the diphosphate moiety of the donor substrate.

Most of the central domain of CEECAM1 failed to yield any enzymatic activity when combined to GLT25D1. The kinetic analysis of the CEECAM1-MidC construct showed that this region was important for the rate of the galactosyltransferase reaction. Considering the predicted GT-A fold of the GLT25 family proteins, this finding suggests that the central domain is important to associate the two lobes of the active enzymes to encompass the donor and acceptor substrates.

The C-terminal domain of the active GLT25D1 enzyme includes two DXD motifs, which are missing in the corresponding domain of the inactive CEECAM1 protein. Our mutagenesis experiments showed that the first of these two DXD motifs is required for the ColGalT activity of GLT25D1, whereas the second one is dispensable. This fact suggests that the C-terminal domain is also involved in the recognition of acceptor substrates and that critical residues for this function are missing in the CEECAM1 sequence. The importance of the C-terminal domain in the recognition of the donor and acceptor substrates was confirmed by the kinetic analysis performed on the partially active chimeric construct CEECAM1-CterC.

Bacterial glycosyltransferases of the CAZY family GT25, such as Lex2B β1,4-glucosyltransferase and LpsB β1,2-galactosyltransferase [26], [27], share structural similarity with the C-terminal part of GLT25D1 between amino acids 344 and 622. These bacterial enzymes are involved in lipopolysaccharide biosynthesis and add hexoses in the oligosaccharide extension. In spite of this structural similarity as seen with the program PROMALS3D, the bacterial Lex2B and LpsB glycosyltransferases also present differences to GLT25D1 since they lack the first DXD motif and the RMQV sequence, which are essential for the ColGalT activity.

The CEECAM1 protein was first described as a cerebral adhesion protein [15], yet the CEECAM1 gene is widely transcribed in the nervous system and in several secretory tissues such as salivary glands, pancreas, liver and placenta [10]. The presence of a potential ER retention signal at the end of the CEECAM1 protein suggests that this protein primarily localizes to the ER, like the active collagen galactosyltransferases GLT25D1 and GLT25D2. Our present study showed that the lack of ColGalT activity of CEECAM1 is related to multiple changes in the protein sequence when compared to GLT25D1 and GLT25D2. However, we cannot exclude at this stage that CEECAM1 does function as a glycosyltransferase that recognizes substrates different from collagen. The presence of potential *CEECAM1* orthologous genes in all mammalian genomes would argue against *CEECAM1* as representing a pseudo-gene or being the consequence of a late gene duplication event specifically found in the human genome.

Taken together, our results provided insights on the functional organization of the GLT25D1 ColGalT by showing that two regions of the protein are essential for the enzymatic activity. Our work also showed that the inactivity of CEECAM1 as a ColGalT relies on multiple amino acid changes, thus suggesting different substrate specificity or different biological activity. The determination of the exact structure of mammalian GLT25 proteins or of homologous bacterial proteins by crystallization will clarify the structural basis of the CEECAM1 functions.

## Materials and Methods

### Materials

Collagen type I from bovine Achilles tendon, UDP-Gal and the anti-Flag M2 monoclonal antibody were from Sigma-Aldrich (Buchs, Switzerland). UDP-[^14^C]Gal was from GE Healthcare (Glattbrugg, Switzerland). Restriction enzymes and DNA modifying enzymes were from New England Biolabs (BioConcept, Allschwil, Switzerland).

### Generation of chimeric proteins GLT25D1-CEECAM1

Full length cDNA fragments were subcloned into pFastBac1-Flag baculovirus transfer vector [28] to construct Flag-tagged GLT25D1 and Flag-tagged CEECAM1 expression plasmids. Chimeric GLT25D1-CEECAM1 constructs were created by replacing parts of the GLT25D1 cDNA sequence by the corresponding CEECAM1 sequence using restriction sites conserved in both genes (Fig. S1). For the CEECAM1-Nter chimeric protein, the restriction sites EcoRI at cDNA position 57 and NcoI (position 796) were used. To create the CEECAM1-Mid and CEECAM1-Cter chimeric proteins, GLT25D1 DNA was digested with PspXI and XbaI, thereby releasing a 1698 bp fragment that was subcloned into pBluescript-SKII (Agilent Technologies, Basel, Switzerland) to create pBluescript-GLT25D1-short. Then, the NcoI-Tth111I and Tth111I-XbaI fragments from GLT25D1 were replaced respectively by the corresponding CEECAM1 sequence using identical restriction sites, thus yielding the constructs pBluescript-CEECAM1-Mid-short and pBluescript-CEECAM-Cter-short. These two new constructs were digested by PspXI and XbaI to subclone the 1698 bp fragments into pFastBac-GLT25D1, yielding the constructs CEECAM1-Mid and CEECAM1-Cter.

For the chimeric proteins CEECAM1-MidA/B/C/D and CEECAM1-CterA/B/C/D/E, restriction sites were introduced by PCR to generate silent mutations without impact on the amino acid sequence. For chimeric proteins CEECAM1-MidA/B/C/D, PCR was performed using pBluescript-GLT25D1-short as template and forward and reverse primers listed in Table 2 and Figure S1. These PCR products were digested and cloned back into pBluescript-CEECAM1-Mid-short, previously linearized using the same enzymes (Table 2, Fig. S1). The pBluescript-based constructs were digested by PspXI and XbaI for cloning back into pFastBac-GLT25D1 as described above, yielding the final constructs CEECAM1-MidA/B/C/D.

**Table 2:** PCR primers used to generate chimeric GLT25D1-CEECAM1 proteins.

CEECAM1 chimeric construct	Plasmid template	Primer	Oligonucleotide sequence (5′-3′)	Restriction site
MidA	pBluescript-GLT25D1-short	For	GCAGTTCCCATGGTGCACTC	NcoI
		Rev	GCAACACGTGCATCTGAACCTCTGCCTGCTTGC	PmlI
MidB	pBluescript-GLT25D1-short	For	GCAGTTCCCATGGTGCACTC	NcoI
		Rev	GCAAACTAGTACCTCCAGCTGCACATGCATG	SpeI
MidC	pBluescript-GLT25D1-short	For	GCAACACGTGTGCAACAAGGAGGAGTACG	PmlI
		Rev	AGCCAGGCAGCATCTGGATC	BamHI
MidD	pBluescript-GLT25D1-short	For	GCAAACTAGTGAAGCACCCGCCCGCAGAGC	SpeI
		Rev	AGCCAGGCAGCATCTGGATC	BamHI
CterA	pBluescript-GLT25D1-short	For	GCAATGGCCACGCAACCTGCATGCCTTC	MscI
		Rev	GCGGCCGCTCTAGAGGCCAC	XbaI
CterB	pBluescript-CEECAM1-Cter-short	For	TGAGGCCGAGAGCTTCATGC	NsiI
		Rev	GCAAGGCGCCCGCCAGACGCAGGGCATAG	KasI
CterC	pBluescript-CEECAM1-Cter-short	For	TGAGGCCGAGAGCTTCATGC	NsiI
		Rev	GCAAGCTCAGGAAGCAGCCCACCTCGCC	Bpu10I
CterD	pBluescript-CEECAM1-Cter-short	For	GCAAGGCGCCCGCAAGCTGCTGGCCTCAC	KasI
		Rev	CGGCCGCTCTAGAGTAGTGGCCTG	XbaI
CterE	pBluescript-CEECAM1-Cter-short	For	GCAACCTGAGCCATTACTCCATCTGGGAAGAGG	Bpu10I
		Rev	GCAAGCTCAGGAAGCAGCCCACCTCGCC	KasI

Plasmids used for PCR amplification are indicated for each constructs with the restriction sites used for cloning. Sequences are shown for the forward and reverse PCR primers, respectively. Bases marked in bold represent positions changed to create new restriction sites. Underlined bases mark the restriction sites used for cloning and dotted underlined bases show the partial restriction sites used for cloning. The BamHI restriction site used in MidC and MidD constructs is present at the 3′-end of the PCR product and not in the PCR primer.

For the chimeric protein CEECAM1-CterA, PCR was performed on pBluescript-GLT25D1-short DNA using the primers given in Table 2. The PCR product was digested by MscI and XbaI (Fig. S2) and cloned back into pBluescript-CEECAM1-Cter-short opened at the same restriction sites. For chimeric proteins CEECAM-CterB/C/D/E, PCR was done on pBluescript-CEECAM1-Cter-short DNA using the corresponding forward and reverse primers listed in Table 2. After restriction digestion (Fig. S2), CEECAM1 fragments were cloned back into pBluescript-GLT25D1-short, previously digested by the same enzymes. Finally, all engineered pBluescript DNA were digested with PspXI and XbaI and introduced into pFastBac-GLT25D1 as described above, yielding the constructs CEECAM1-CterA/B/C/D/E. The integrity of the constructs generated by PCR was verified by DNA sequencing (Microsynth, Balgach, Switzerland).

### Mutagenesis

Site directed mutagenesis of GLT25D1 and chimeric GLT25D1-CEECAM1 constructs was performed by QuickChange site-directed mutagenesis [29]. The corresponding primers and DNA templates are shown in Table 3. The introduction of the appropriate mutations was confirmed by DNA sequencing (Microsynth). The mutant constructs are named according to the amino acids substituted and their respective position on the protein sequence where the start methionine of GLT25D1 counts as position one.

**Table 3:** Sense strand oligonucleotides used for site-directed mutagenesis.

Plasmid template	Mutations	Oligonucleotide sequence (5′-3′)
GLT25D1	D166A-D168A	CATCCTGTTTGTAGCGGCCGCCAACCTGATCCTCAACCC
GLT25D1	P292N	GGAGTACGGATTCTTGAACGTTCCATTGCGCGCCCACAGC
GLT25D1	D336S	CCCACCAAGACACCGAGCAAGATGGGCTTCG
GLT25D1	D461A-D463A	GGAGGGCCTGGCCTGGGCCCTCATCTATGTGG
GLT25D1	D585A-D587A	CGTCAAGACCGCCTGGGCCCGCGCCAAGTCC
CEECAM1-CterB	S461D	GGAGGCAGAGAAACTGGATTGGGACCTGATCTACC
CEECAM1-CterB[S461D]	Q471R-V472M-N473Q-P474V	GATCTACCTCGGACGGAAGCGCATGCAGGTTGAGAAGGAGACGGCCGTGG

Sequences show the sense strand oligonucleotides of each complementary pair of primers used for site-directed mutagenesis. Underlined bases mark the introduced mutations.

### ColGalT assays

Recombinant baculoviruses were produced in *Spodoptera frugiperda* Sf9 cells as described previously [30]. Galactosyltransferase assays were performed as described by Schegg *et al.* [10]. Briefly, 2.10^6^ infected Sf9 cells were lyzed in 50 mM TrisHCl pH 8, 150 mM NaCl, 1% Triton X100 and protease inhibitors (Complete, Roche, Rotkreuz, Switzerland) for 15 min on ice. Insoluble material was precipitated at 14,000×g for 20 min at 4°C. Assays were performed using 10 µl of postnuclear supernatant, 5 mg/ml of denatured collagen type I from bovine Achilles tendon, 60 µM UDP-Gal spiked with 50,000 cpm UDP-[^14^C]Gal, 10 mM MnCl_2_, 20 mM NaCl, 50 mM morpholinepropanesulfonic acid pH 7.4 and 1 mM DTT. Reactions were incubated for 3 h at 37°C then stopped by addition of 500 µl of 5%TCA/5% phosphotungstic acid. The precipitated assay products were applied on glass fiber filters (Whatman, Sigma-Aldrich), washed with 10 ml of 50% ethanol, dried for 30 min and counted in a scintillation β-counter (Packard).

### Western Blotting

After determination of protein concentration, samples corresponding to 10 µg proteins were denatured at 95°C, loaded on 8% SDS-PAGE, and transferred to PVDF membranes (Millipore, Zug, Switzerland). Flag proteins were detected using an anti-Flag M2 monoclonal antibody (dilution 1∶2,000) (Sigma-Aldrich), then by a secondary antibody, anti-mouse peroxidase conjugate (dilution 1∶10,000) (Sigma-Aldrich), followed by detection using SuperSignal West Pico chemiluminescent substrate (Thermo Scientific, Lausanne, Switzerland).

### Alignment and modeling of protein structure

Protein sequences corresponding to human GLT25D1 (Swiss-Prot:Q8NBJ5), human GLT25D2 (Swiss-Prot:Q81IYK4) and human CEECAM1 (Swiss-Prot:Q5T4B2) were used as input for the PROMALS3D multiple sequence and structure alignment tool (http://prodata.swmed.edu/ promals3d/promals3d.php) [18]. An identity threshold of 0.6 was chosen and other settings were set as default. Sequences were aligned according to predicted α-helices and β-strands. Protein homology detection of the three proteins was done using the HHpred program based on the pdb70 database (http://toolkit.tuebingen.mpg.de/hhpred) [19]. Multiple sequence alignment was done using ClustalW2 program (http://www.ebi.ac.uk/Tools /msa/clustalw2/) [31].

## Supporting Information

Figure S1 **Schematic representation of the PCR fragments used to generate chimeric CEECAM1-MidA/B/C/D constructs.** Segments of GLT25D1 and CEECAM1 are marked in light grey and dark grey, respectively. The full length GLT25D1 cDNA is represented at the top with the relative positions of the restriction sites used for cloning. The partial region of pBluescript-CEECAM1-Mid-short with the corresponding restriction sites is shown below with the PCR products and primers (arrows) including restriction sites used for cloning.
(TIF)

Figure S2 **Schematic representation of the PCR fragments used to generate chimeric CEECAM1-CterA/B/C/D/E constructs.** The top panel shows the full length GLT25D1 cDNA with the restriction sites used for cloning. Below the C-terminal domain of CEECAM1-Cter-short and the CterA construct are represented as in supplemental figure S1. The bottom panel shows the C-terminal domain of GLT25D1 with the relative positions of the restriction sites used for cloning. Below, the CterB/C/D/E constructs are represented as in supplemental figure S1.
(TIF)
