# Supplementary figures and images for: Identification of Domains and Amino Acids Essential to the Collagen Galactosyltransferase Activity of GLT25D1
Source: PLoS One. 2011 Dec 21;6(12):e29390. doi: 10.1371/journal.pone.0029390 (PMC3244457; doi:10.1371/journal.pone.0029390)

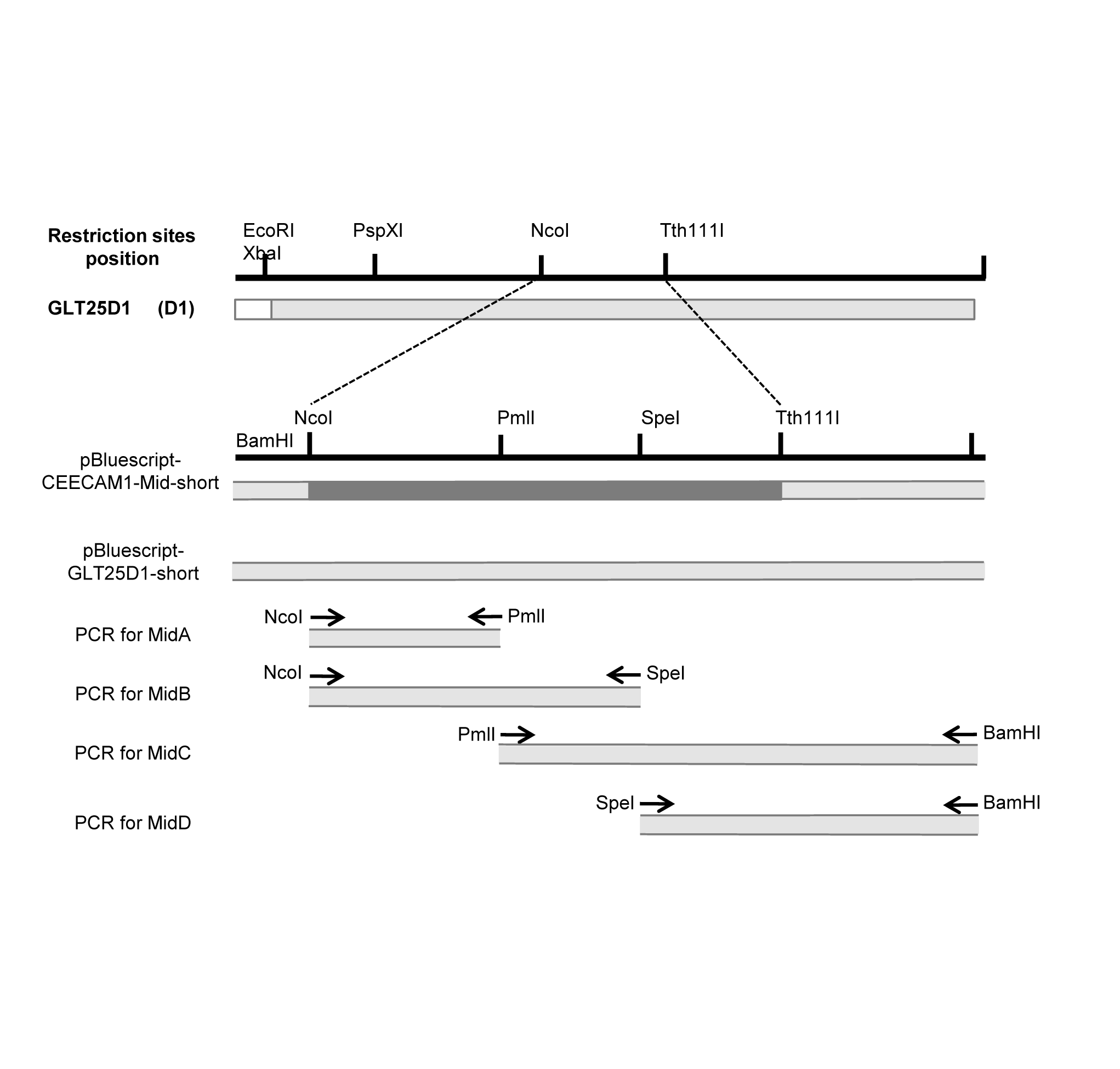

Supplement: Figure S1 — Schematic representation of the PCR fragments used to generate chimeric CEECAM1-MidA/B/C/D constructs. Segments of GLT25D1 and CEECAM1 are marked in light grey and dark grey, respectively. The full length GLT25D1 cDNA is represented at the top with the relative positions of the restriction sites used for cloning. The partial region of pBluescript-CEECAM1-Mid-short with the corresponding restriction sites is shown below with the PCR products and primers (arrows) including restriction sites used for cloning. (TIF) [file pone.0029390.s001.tif]

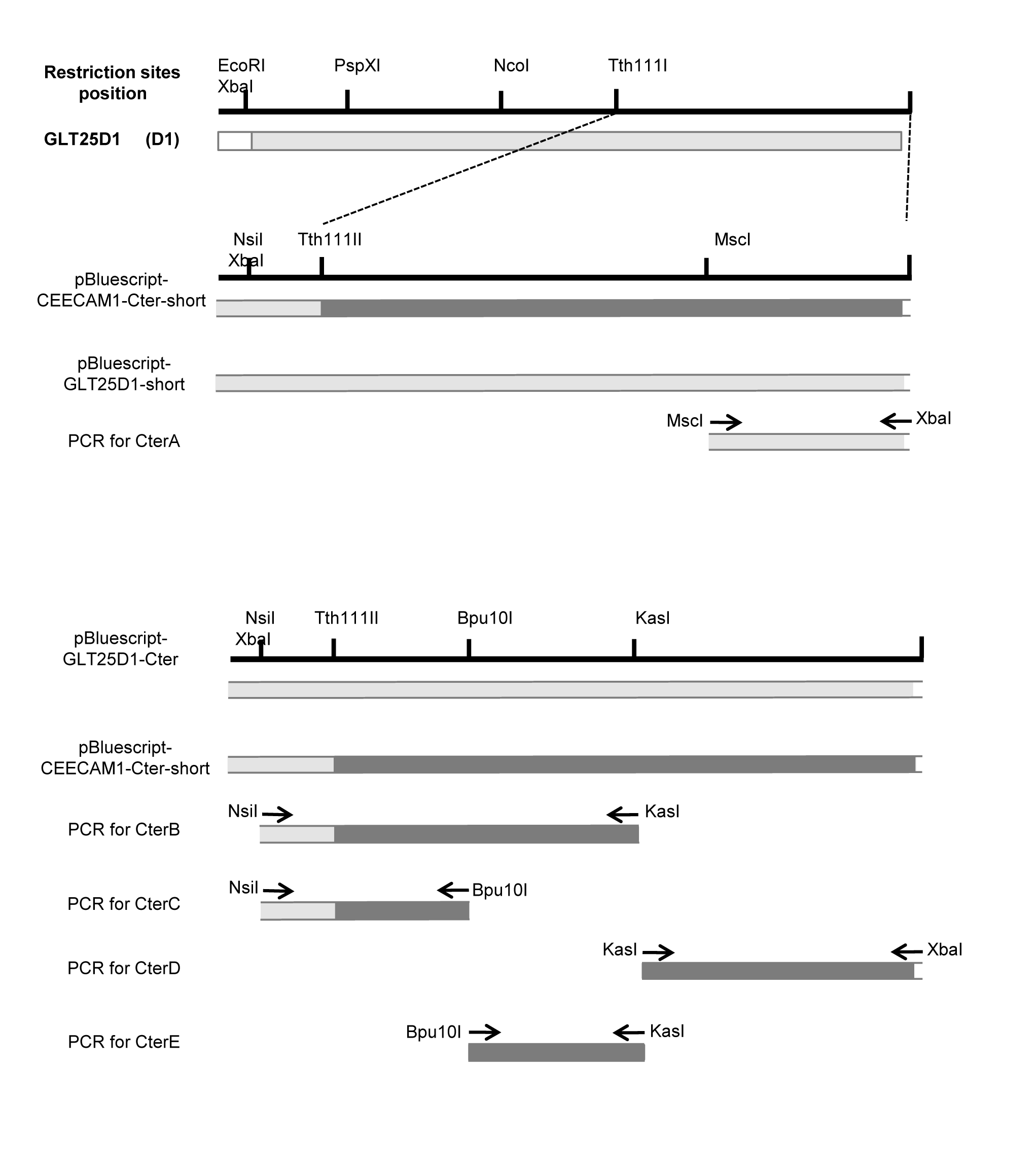

Supplement: Figure S2 — Schematic representation of the PCR fragments used to generate chimeric CEECAM1-CterA/B/C/D/E constructs. The top panel shows the full length GLT25D1 cDNA with the restriction sites used for cloning. Below the C-terminal domain of CEECAM1-Cter-short and the CterA construct are represented as in supplemental figure S1. The bottom panel shows the C-terminal domain of GLT25D1 with the relative positions of the restriction sites used for cloning. Below, the CterB/C/D/E constructs are represented as in supplemental figure S1. (TIF) [file pone.0029390.s002.tif]
